# Hemopneumothorax Secondary to Pleural Endometriosis in a Woman of Childbearing Age: A Rare Presentation

**DOI:** 10.7759/cureus.71202

**Published:** 2024-10-10

**Authors:** Leonor Ávila, Helena Leandro, Rita Silva, Rui Mendes, Fátima Coelho

**Affiliations:** 1 General Surgery, Unidade Local de Saúde Lisboa Ocidental, Lisbon, PRT

**Keywords:** chest drainage, endometriosis, hemopneumothorax, hemothorax, pleural endometriosis, thoracic endometriosis

## Abstract

Pleural endometriosis is a rare condition characterized by the presence of endometrial tissue outside the uterus, involving the pleura. This atypical condition can be asymptomatic or cause respiratory symptoms such as chest pain, dyspnea, and hemothorax. We present a clinical case of a 32-year-old woman who presented to the emergency room with hemothorax due to pleural endometriosis.

## Introduction

Endometriosis is a condition characterized by the presence of ectopic endometrial tissue, commonly within the pelvic region, but can occur in extrapelvic sites, including the pleural cavity, bowel, brain, diaphragm, and skin [1]. Endometriosis is a non-cancerous, inflammatory condition influenced by estrogen that impacts females during their premenstrual, reproductive, and postmenopausal phases.

Thoracic endometriosis is an uncommon condition that mainly affects young women. Its prevalence in the general population is not well documented, but it has been observed in less than 1% of women undergoing pelvic surgery for suspected or confirmed pelvic endometriosis [2, 3]. Higher rates are seen in individuals with primary spontaneous pneumothorax, ranging from 3% to 6%. For those with recurrent pneumothorax or cases requiring surgery, the rates increase to between 6% and 20%, while in cases of catamenial pneumothorax, the rates can be as high as 65% to 89%. Depending on its manifestation, it can be asymptomatic or cause severe respiratory symptoms, which can be life-threatening [4-8]. 

In this report, we present a clinical case involving a 32-year-old woman who arrived at the emergency room with hemothorax, caused by pleural endometriosis, and required prompt attention and medical care. This case emphasizes the challenges and vital significance of timely diagnosis, as well as the necessity for proactive management to reduce the risk of serious complications associated with this condition.

The patient provided informed consent for the use of her clinical information and medical photographs, and her privacy and confidentiality were strictly maintained throughout the process.

## Case presentation

**Figure 1:**
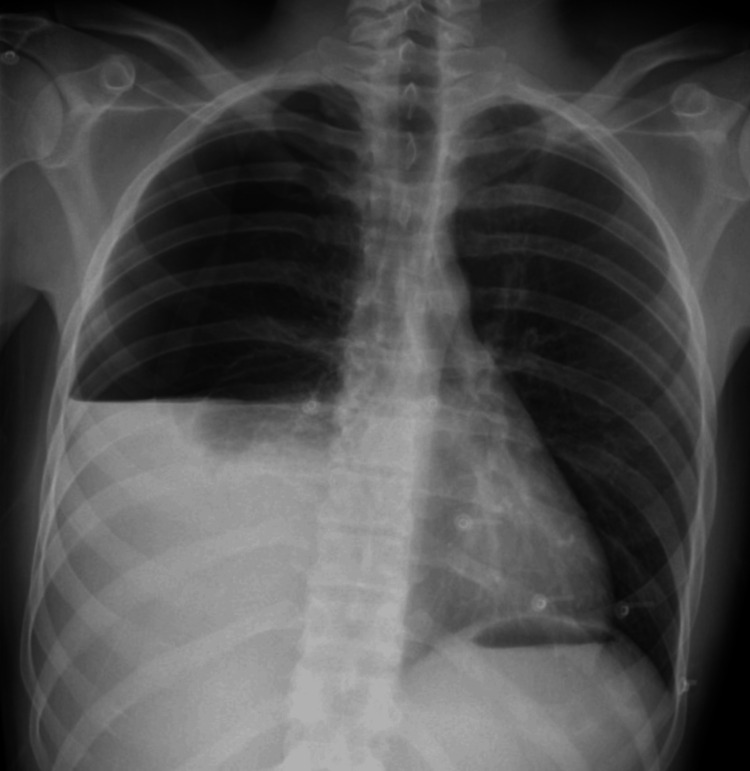
Chest X-ray demonstrating the hemothorax

**Figure 2:**
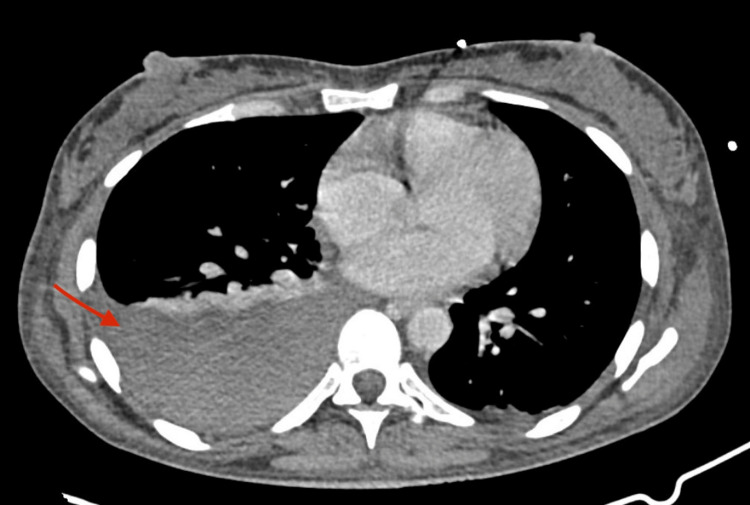
Thoracic CT scan (axial plane) demonstrating the hemothorax (red arrow)

**Figure 3:**
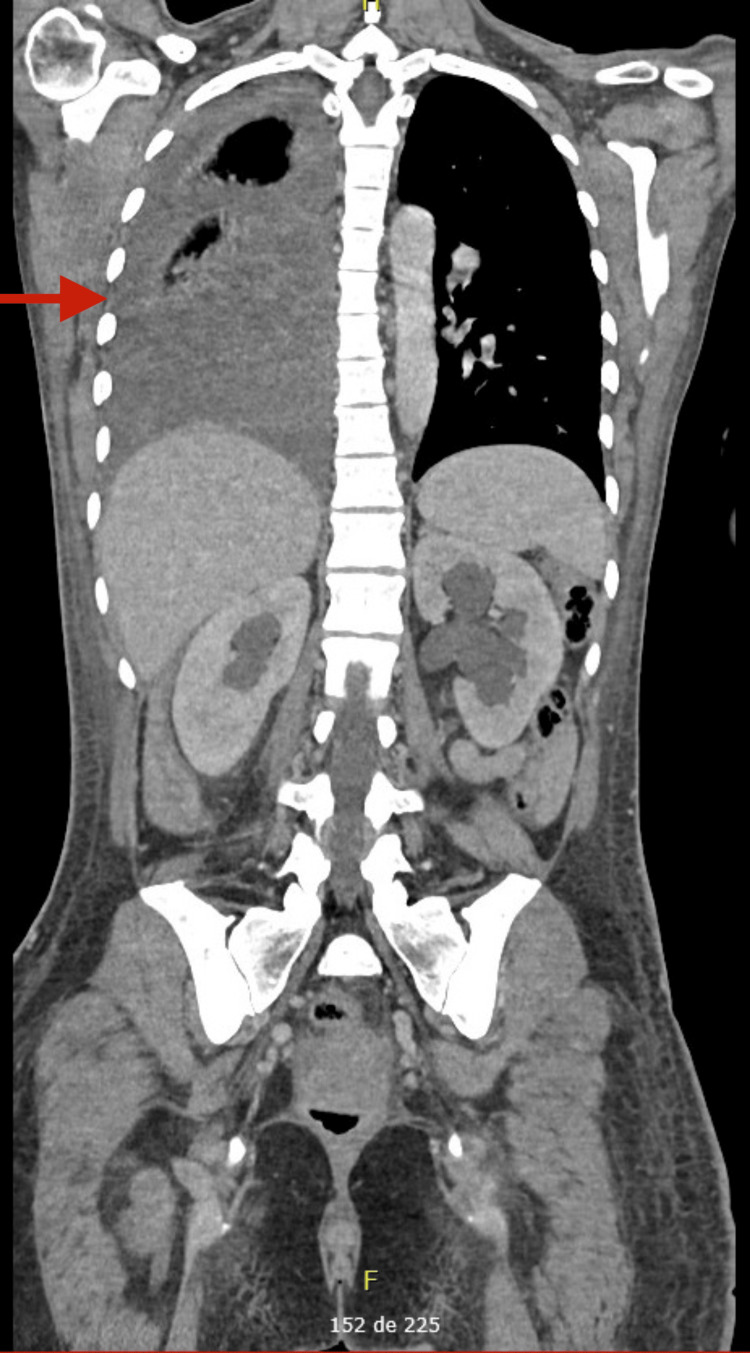
Thoracic CT scan (coronal plane) demonstrating the hemothorax (red arrow)

A 32-year-old nulliparous woman with a known history of endometriosis diagnosed by previous laparoscopy with excision of endometriosis' implants on the peritoneal cavity, presented to the emergency department with acute onset right chest pain, dyspnea, and a history of recent menstruation. She reported a recurrent right-sided pleuritic plain that had worsened over the past 48 hours. The patient denied any history of trauma or other significant medical conditions. Physical examination revealed decreased breath sounds on the right side and dullness to percussion. A chest X-ray and computed tomography (CT) scan demonstrated a large right-sided hemothorax. Hemodynamic instability with tachycardia (heart rate (HR) 110 beats per minute (bpm)) and hypotension (85/70 mmHg) was noted. Still, normal values of oxygen saturation (96%) and partial pressure of oxygen (pO_2_ 78.2 mmHg) were present (Figures 1-3).

**Figure 4:**
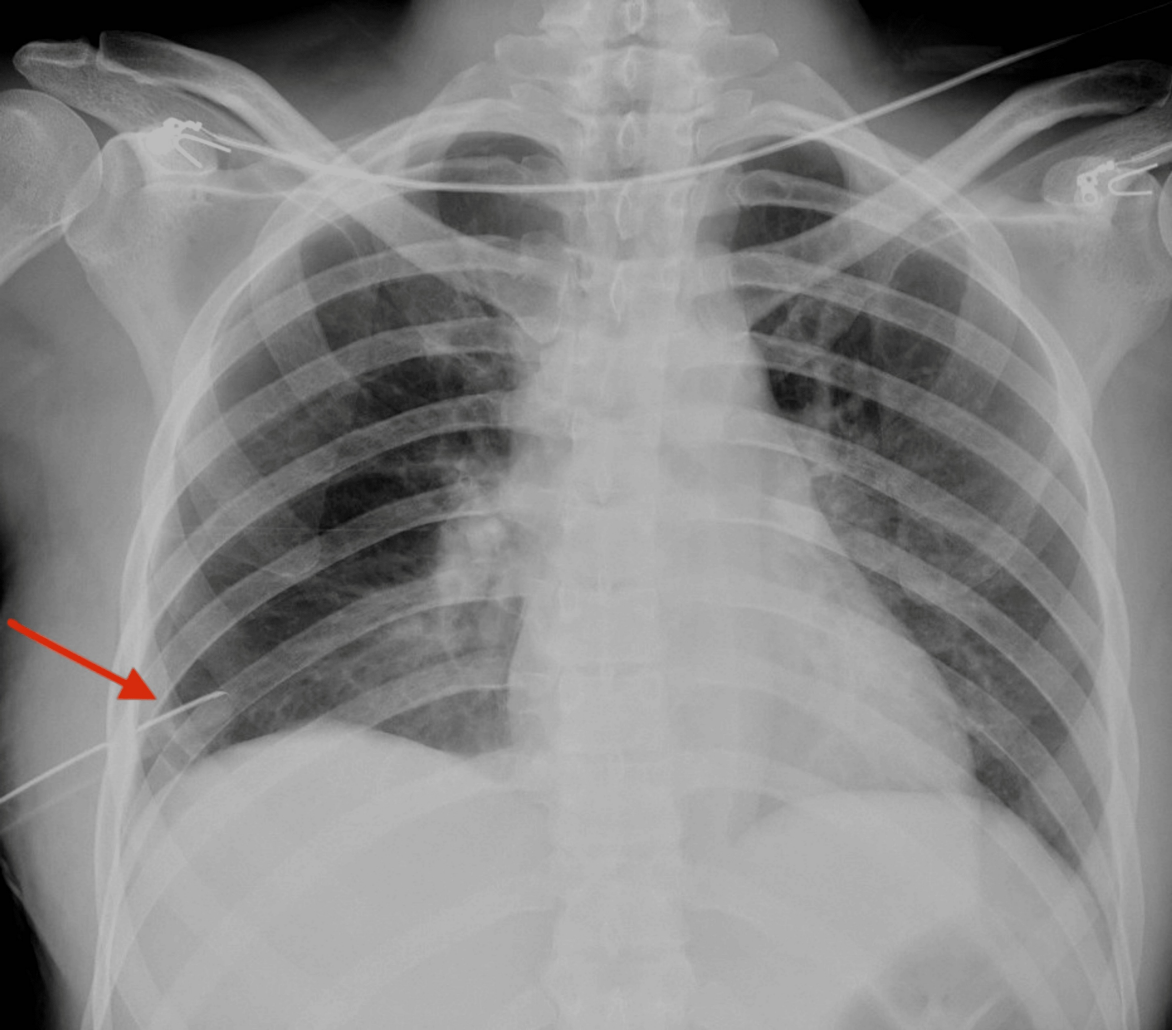
Chest X-ray demonstrating the chest tube The red arrow demonstrates the chest tube in the sixth intercostal space.

Laboratory investigations showed a decreased level of hemoglobin (6 g/dL) consistent with active bleeding into the pleural space; she also presented with leukocytosis (13.7 x 10^9^/L) and an increased level of C-reactive protein (6.5 mg/dL). Regarding renal function, it was evident an acute renal failure, with a serum creatinine level of 1.40 mg/dL with normal urea levels. The blood coagulation parameters were within normal ranges. Immediate resuscitation, with transfusion of one unit of red blood cells, and chest tube placement were performed, which revealed frank blood (850 mL), and pleural fluid analysis confirmed the presence of endometrial cells (Figure 4).

A diagnosis of hemothorax secondary to pleural endometriosis was made based on clinical presentation (woman of childbearing age with acute respiratory symptoms), radiological findings (evidence of hemothorax on radiologic exams), and the presence of endometrial cells in pleural fluid. 

**Figure 5:**
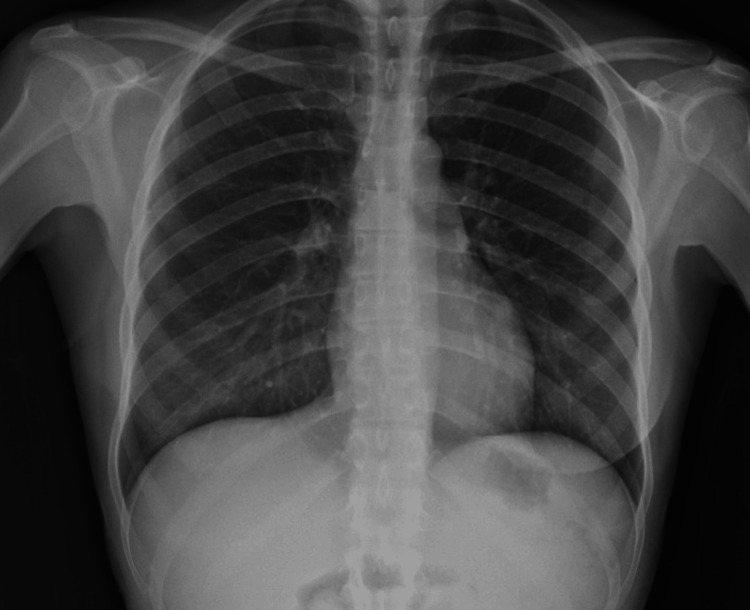
Chest X-ray at the discharge demonstrating the resolution of the hemothorax

Hormonal therapy with a gonadotropin-releasing hormone (GnRH) agonist was initiated promptly to induce amenorrhea and reduce endometrial tissue activity. The chest tube was removed once the hemothorax resolved (last output of 50 mL/day), and the patient was discharged after five days with a plan for long-term hormonal therapy and close follow-up, with a gynecology consult, to monitor for recurrence (Figure 5).

## Discussion

Pleural endometriosis is a rare condition that can occur in isolation or in association with pelvic endometriosis. Several theories have been put forward to explain how thoracic endometriosis develops, some of which overlap with those for pelvic disease. They are as follows: (1) Autotransplantation via retrograde menstruation: Sampson's theory suggests that endometrial tissue can reach the thoracic cavity through retrograde menstruation, where menstrual blood flows backward through the fallopian tubes, leading to the transplantation of endometrial cells into both the peritoneal and thoracic cavities [9]; (2) Micro-embolization/metastasis: Another proposed mechanism is the metastatic spread of endometrial tissue via the venous or lymphatic systems to the lungs [10]. Evidence supporting this includes the discovery of endometrial foci in areas distant from the pelvis and thorax, such as the brain, knee, and eye. Additionally, circulating endometrial cells have been found in 90% of patients with confirmed pelvic endometriosis; (3) Coelomic metaplasia: This theory posits that pluripotent cells can transform into differentiated endometrial tissue, a process known as coelomic metaplasia. Support for this idea comes from the identification of pluripotent cells in the uterine endometrium [11, 12].

The diagnosis of pleural endometriosis is challenging and often requires a high clinical suspicion. Clinical symptoms can range from asymptomatic until pleuritic chest pain or even massive hemothorax, as observed in this case [4-8].

Detailed medical history with special attention to the menses, imaging studies that can reveal endometrial implants, and pleural fluid analysis (demonstrating the presence of endometrial cells and stroma within the pleura) are valuable tools for accurate diagnosis.

The management of patients with thoracic endometriosis frequently involves multiple disciplines, including pulmonologists, thoracic surgeons (surgical resection of endometriotic implants to prevent hemothorax recurrence), and gynecologists. In this particular case, after discharge, the patient was oriented to a thoracic surgery appointment with the goal that she would subsequently undergo surgery to remove the thoracic endometriosis implants. Because of the multiple different disciplines involved, good communication among clinicians is critical for successful outcomes. Nevertheless, treatment choice should be individualized and discussed with the patient, considering her reproductive desires [8].

## Conclusions

Pleural endometriosis is a rare manifestation of endometriosis that can be silent or present with severe symptoms such as hemothorax. While thoracic endometriosis can sometimes occur isolated, it typically presents alongside significant endometriosis affecting the reproductive, genitourinary, and gastrointestinal systems. In patients diagnosed with thoracic endometriosis, approximately 50% to 84% also have pelvic endometriosis. Early diagnosis and appropriate treatment are essential to prevent complications and ensure the patient's complete recovery. This case report highlights the importance of considering pleural endometriosis in patients with acute respiratory symptoms in women of childbearing age, especially in the presence of known endometriosis, and underscores the need for a multidisciplinary approach to effective management. Close follow-up is essential to monitor for recurrence.
